# Warning labels on alcoholic beverage containers: a pilot randomized experiment among young adults in Mexico

**DOI:** 10.1186/s12889-023-16069-w

**Published:** 2023-06-15

**Authors:** Nancy López-Olmedo, Karla Muciño-Sandoval, Francisco Canto-Osorio, Adriana Vargas-Flores, Alai Quiroz-Reyes, Arturo Sabines, Miguel Malo-Serrano, Sergio Bautista-Arredondo, MArantxa Colchero, Tonatiuh Barrientos-Gutiérrez

**Affiliations:** 1 Center for Population Health Research, National Institute of Public Health, Cuernavaca, Morelos Mexico; 2 Center for Health Systems Research, National Institute of Public Health, Cuernavaca, Morelos Mexico; 3 Pan American Health Organization in Mexico, Mexico City, Mexico

**Keywords:** Alcohol, Health warning label, Randomized controlled trial, Pilot study, Mexico

## Abstract

**Background:**

Little is known about the potential impacts of visible and up-to-date health warning labels on alcoholic beverage containers on a range of outcomes in low- and middle-income countries. We conducted an experimental study to test the potential impacts of visible health warning labels (on the principal panel of the package) on thinking about health risks, product attractiveness, visual avoidance, and intention to change alcohol use among students in Mexico aged 18–30 years.

**Methods:**

A double-blind, parallel-group, online randomized trial was conducted from November 2021 to January 2022 in 11 states in Mexico. In the control group, participants were presented with the image of a conventional beer can with a fictional design and brand. In the intervention groups, the participants observed pictograms with a red font and white backgrounds (health warning label in red—HWL red) or with a black font and yellow backgrounds (health warning label in yellow—HWL yellow), located at the top, covering around one-third of the beer can. We used Poisson regression models -unadjusted and adjusted for covariates- to assess differences in the outcomes across study groups.

**Results:**

Using intention-to-treat analysis (*n* = 610), we found more participants in groups HWL red and HWL yellow thought about the health risks from drinking beer compared to the control group [Prevalence Ratio (PR) = 1.43, CI95%:1.05,1.93 for HWL red; PR = 1.25, CI95%: 0.91, 1.71 for HWL yellow]. A lower percentage of young adults in the interventions vs control group considered the product attractive (PR 0.74, 95%CI 0.51, 1.06 for HWL red; PR 0.56, 95%CI 0.38, 0.83 for HWL yellow). Although not statistically significant, a lower percentage of participants in the intervention groups considered buying or consuming the product than the control group. Results were similar when models were adjusted for covariates.

**Conclusions:**

Visible health warning labels could lead individuals to think about the health risks of alcohol, reducing the attractiveness of the product and decreasing the intention to purchase and consume alcohol. Further studies will be required to determine which pictograms or images and legends are most contextually relevant for the country.

**Trial registration:**

The protocol of this study was retrospectively registered on 03/01/2023: ISRCTN10494244.

**Supplementary Information:**

The online version contains supplementary material available at 10.1186/s12889-023-16069-w.

## Background

Alcohol is a risk factor for more than 200 health problems, yet it is the most widely consumed psychoactive substance globally [1, 2]. In Mexico, alcohol consumption in the past 30 days among adults (aged 18–65 years) increased from 35% in 2011 to 40% in 2016, while binge drinking in the 30 days increased from 14 to 22% [3]. To address this public health issue, the World Health Organization (WHO) Global Strategy to Reduce the Harmful Use of Alcohol includes, among other policy recommendations, labeling alcoholic beverages to raise awareness about the negative consequences of alcohol use [4]. Moreover, the WHO Draft Action Plan 2022–2030 proposes, among other actions, the development and implementation of warning labels [5].

Warning labels on alcoholic beverages are currently implemented in 47 countries [6, 7]; however, label characteristics are heterogeneous, and their use is not necessarily mandatory. Warnings that are small in size, and on the sides or back of the package are standard, providing information about the general health risks of alcohol consumption, risks during pregnancy, or when driving a motor vehicle [7]. In general, labels fail to communicate up-to-date evidence, such as the link between alcohol consumption and 7 types of cancer. In 2019, 67% of US adults reported being unaware that alcohol is a risk factor for cancer [8].

The regulation on health warning labels for alcoholic beverages in Mexico is also limited. The regulation has not been updated since 2014 and establishes that alcohol containers must include a cautionary legend (*abuse in the consumption of this product is harmful to health*) and at least one small pictogram warning about alcohol use during pregnancy, consumption while driving, or prohibiting alcohol sales to minors. The regulation does not specify where the legend and pictograms must be located, so they are usually shown on the side or back of the container. In the case of beverages with low alcohol content (2.0% to 6.0%), such as beer, packages are only required to include the legend *18* + *Not for sale to minors*, and even the warning can be placed only on the bottle cap [9], even though this product is the most consumed in Mexico [10].

Evidence from randomized trials –mainly online studies from the United Kingdom, Australia, and Canada– suggests that health warning labels on alcoholic beverages that are visible and up-to-date can impact a range of outcomes beyond raising awareness, including product attractiveness, visual avoidance, and intention to change consumption [11, 12]. Specifically, health warning labels that contain messages with negative frames accompanied by images (e.g. pictograms) can be more effective [11, 12]. In addition to the "appeal to fear", negative messages can elicit an emotional response that may lead those exposed to them to describe the consequences of alcohol use [13]. Also, as in other products such as tobacco and sugar-sweetened beverages, health warning labels on alcoholic beverages can counteract the attractiveness of their designs [14, 15]. Finally, based on the theory of prospect frames (gain- versus loss-framed messages) applied to health warning labels on alcoholic beverages, messages with negative frames can increase defensive avoidance (e.g. do not put effort into reading them) and behavioral intentions in line with the message [16].

To our knowledge, experimental studies have not been conducted in Mexico to evaluate the impact of health warning labels on raising awareness about health risks, attitudes, and behavioral intentions. Studies are needed in low- and middle-income countries where sociodemographic characteristics and alcohol consumption patterns may differ. This study aimed to conduct a randomized experiment to assess the potential impact of visible health warning labels (located at the top of the front of the package) on thinking about health risks, product attractiveness, visual avoidance, and intention to change the consumption of the alcoholic beverage presented among young adults in Mexico. This is the first study testing alcohol warning labels with varying health messages recommended by the WHO Europe [17].

## Methods

### Trial design

We conducted a pilot randomized experiment on young adults in Mexico. Participants were assigned to one of three parallel groups via the LimeSurvey platform through simple randomization (1:1:1 allocation ratio). The participants and researchers were kept blinded to the allocation, except for the data analyst.

### Participants

Eligible participants were males and females in Mexico between 18 to 30 years old, studying at public and private schools of upper-middle and higher level, who had access to a smart mobile device or a computer with an internet connection. We excluded participants who reported not having consumed beer in the last 12 months. We considered this exclusion criterion since we used images of a beer can, the most consumed alcoholic beverage in Mexico [10]. We also excluded females who were currently pregnant or breastfeeding because they could have changed their alcohol intake patterns. We estimated a sample size of 1,680 participants, randomized into three groups of 560 each, as described below, based on the results of a similar study in the United Kingdom [18, 19], and considering a power of 0.8, an effect design of 2.2 and an alpha value of 0.0167 (value of 0.05 adjusted for multiple comparisons).

Based on the 2011 National Addictions Survey, we stratified the sample by region (north-central, northwestern, northeastern, west, central, Mexico City, central-south, and south) to represent the variability in alcohol consumption across the country [20]. The sample size was proportional to the total population aged 20 to 30 years in each region, according to the 2020 Census. We decided to recruit participants from at least one state per region, except for central and central-south, where we recruited participants in 3 and 2 states, respectively, since more young adults live in these regions than others.

Officials from the Pan American Health Organization in Mexico contacted local authorities (officials from the local Ministries of Health and Education and officials from public and private schools) from 11 states (Supplementary Table 1) to promote the study among upper-middle and higher-level public and private schools. Researchers at the National Institute of Public Health also announced the study in schools from selected states.

Ethics approval for this study was obtained from the *Consorcio de Investigación en Salud* (CISIDAT) Research Ethics Committee, Mexico (reference: FWA 00031322).

### Interventions

#### Health warning labels

**Fig. 1:**
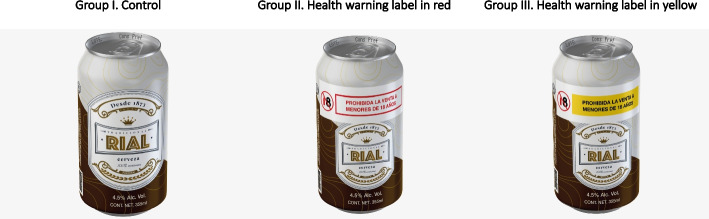
General design of the beer can by experimental condition

For the experimental stimuli, we used images of a conventional beer can (355 mL) with a fictional design and brand. The beer displayed one of three labels on the front panel: I) no label control, II) health warning label in red (HWL red), and III) health warning label in yellow (HWL yellow). In the control condition, participants were shown the beer can with no warning label. In the intervention groups, pictograms and legends were located at the top, covering a little less than one-third of the front of the package as proposed by WHO Europe [17]. In the HWL red group, the legends were presented in red font with white background, also proposed by WHO Europe [17], while the HWL yellow used black font with a yellow background, similar to the warning labels on cigarette packages in Mexico (Fig. 1). Participants assigned to intervention groups were, in turn, randomly assigned to one of eight legends and pictograms as recommended by WHO Europe: 1) *Alcohol consumption may harm the unborn baby*, 2) *Sale to minors under 18 is prohibited*, 3) *Driving under the influence of alcohol is prohibited*, 4) *Alcohol consumption can cause liver cirrhosis*, 5) *Alcohol consumption can cause mental health problems*, 6) *Prohibited from consuming alcohol while operating machinery*, 7) *Alcohol consumption can cause cancer*, 8) *Alcohol consumption can cause dependence* (Supplementary Fig. 1) [17].

### Procedures

The online questionnaire was available from November 9, 2021, to January 17, 2022. The questionnaire included five sections that participants observed in the same order. We describe each section below.

- Section 1. Informed consent. This section included a general study description, confidentiality, data management, risks and benefits, and contact details. If participants agreed to participate, they were asked for an email to send a copy of their informed consent.
- Section 2. Filter questions. Participants with at least one exclusion criterion or who did not answer at least one question of this section ended their participation.
- Section 3. Sociodemographic characteristics. This section included the following indicators: educational level [1) pursuing a bachelor’s degree, 2) pursuing a postgraduate degree], field of specialization [1) physics, mathematics, or engineering, 2) biological or health sciences, 3) social sciences, 4) arts or humanities], educational and employment status [1) studying, 2) studying and working, 3) studying and looking for a job] indicators of socioeconomic status (housing floor materials and assets) and state of residence. Once the participants answered this section, the LimeSurvey platform allocated them to one of the three study groups.
- Section 4. Main study questions. An image of a can of beer with a randomly assigned label according to the experimental arms was presented, accompanied by questions about perceived risk, intention to change the consumption of the alcoholic beverage presented, product attractiveness, and visual avoidance (avoid seeing the can of beer).
- Section 5. Alcohol consumption. This section included questions related to the frequency and quantity of alcohol consumption.

### Outcomes

We measured the ability of the health warning labels on four items: 1) thinking about health risks, 2) overall product attractiveness, 3) visual avoidance, and 4) intention to change the purchase and consumption of the alcoholic beverage presented. We evaluated each item using questions previously developed as references. Specifically, we used the questions developed by Clarke et al. for thinking about health risks, attractiveness by Al_hamandi & Smith, visual avoidance by Blackwell et al., and Pechey et al. for intention to change [18, 21–23]. In October 2021, we proved the adequacy of the questions in a small sample of volunteers with similar characteristics to the study sample (*n* = 43) and made the modifications accordingly. Questions are described in Table 1.

**Table 1:** Main study questions

Question	Answer options
*How attractive do you find the product shown?*	Not attractive at all
	Little attractive
	Attractive
	Very attractive
	I do not wish to reply
*According to the image shown, order the following characteristics* (color and design, brand, alcohol volume, and net content in all study groups, and the health warning label in intervention groups) *of the beer can from "The one that most attracts your attention" to the one that "Catches your attention less"*	NA
*How likely are you to buy it?*	Very unlikely
	Unlikely
	Somewhat likely
	Likely
	Very likely
	I do not wish to reply
*Looking at the image shown, did you avoid seeing the label?* With “label”, we referred to the brand in the control group and the health warning label in the intervention groups	Yes
	No
	I do not wish to reply
*How likely are you to consume it?*	Very unlikely
	Unlikely
	Somewhat likely
	Likely
	Very likely
	I do not wish to reply
*Did you think about the health risks involved in drinking it?*	Not at all
	A little
	Somewhat
	Very much
	I do not wish to reply

Due to the low response in some of the categories in questions of interest and to analyze the responses unadjusted and adjusted for covariates using log-binomial models that directly model the prevalence ratio, [24] we further grouped the responses into two categories as described below.

- *Thought about the health risks involved in drinking it *(very much/somewhat vs a little/not at all)
- *Attractive product* (very attractive/attractive vs somewhat attractive/not at all attractive)
- *Likely to purchase* (very likely/likely/somewhat likely vs unlikely/very unlikely)
- *Likely to consume* (very likely/ likely/somewhat likely vs unlikely/very unlikely)

We grouped the category of *somewhat likely* as “*likely”* since it is expected that categories that include the same phrase (in this case, *very likely*, *likely*, and *somewhat likely*) are highly correlated [25].

### Covariates

Covariates included in the analyses were: socioeconomic level, sex, age, educational level, area of studies, educational and employment status, and frequency of alcohol consumption. Variation in household income is expected among students because they were recruited from public and private schools. Therefore, we estimated a socioeconomic level using principal components analysis applied to households’ assets and characteristics of the house. The first principal component explained 25% of the variability in the sample and was used to calculate the index score for each participant. The index was classified into tertiles representing low, medium, and high socioeconomic status.

The frequency of alcohol consumption was determined using a question previously validated and used in the 2016–2017 National Survey on Drug, Alcohol, and Tobacco Consumption [26]. We reclassified the responses into the following four categories, given the low response in some categories:

1. *At least once a week* includes the categories: three or more times a day, two times a day, once a day, almost every day (5–6 times a week), three or four times a week, and once or twice a week,
2. *At least once a month* includes the categories: approximately once a month and two or three times a month.
3. *At least once in the last 12 months* includes the categories: seven to eleven times in the last 12 months, three to six times in the last 12 months, two times in the last 12 months and once in the last 12 months.
4. *I do not want to answer*

### Statistical analyses

As descriptive analyses, medians and interquartile ranges were calculated for the continuous variables and percentages for the categorical variables, overall and by study group. We first described the prevalence of responses for each question and original categories on the Likert scale, in the total sample, and by study group. Then, we used log-binomial regression models with robust variance errors to assess outcome differences across study groups unadjusted and adjusted for covariates. We determined statistically significant differences between groups if the confidence intervals did not cross the null value. However, as recommended, we also discussed results that provide a broader picture of the results, even though not statistically significant. In the same sense, we did not present p-values given their dichotomous use into “significant” and no “significant”, which is no longer recommended [27]. All statistical analyses were performed using the Stata program version 14.0 (Stata Corp, College Station, TX, USA).

### Post-hoc analyses

We carried out a post-hoc analysis to further explore the potential effect of the health warning label messages on the participants’ responses. Specifically, we analyzed the potential effect of the health warning labels in red and yellow on the participants’ responses by grouping them, given the limited sample by type of legend in the intervention groups. We grouped the health warning labels into three categories: “Prohibitions on sale and consumption”, “Health risks”, and “Mental health problems”. “Prohibitions on sale and consumption” included the health labeling messages *Sale to minors under 18 is prohibited*, *Driving under the influence of alcohol is prohibited*, and *Prohibited from consuming alcohol while operating machinery*. “Health risks” included: *Alcohol consumption may harm the unborn baby*, *Alcohol consumption can cause liver cirrhosis*, and *Alcohol consumption can cause cancer*. Finally, “Mental health problems” included two messages: *Alcohol consumption can cause mental health problems* and *Alcohol consumption can cause dependence*. We conducted log-binomial regression models with robust variances (unadjusted and fully adjusted) to evaluate the potential effect of each group of messages on participants’ responses. We used the “Prohibitions on sale and consumption” as the reference. The pictograms of this category must be included in alcoholic beverages in Mexico, although in a smaller size on the side of beer cans.

## Results

**Fig. 2:**
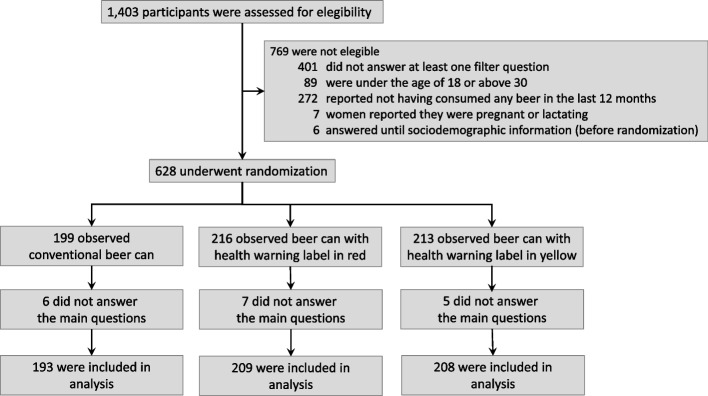
Flow of participants through study

A total of 628 participants were randomized; 199 were allocated to the control group and 216 and 213 to the HWL red and HWL yellow groups, respectively. A total of 6, 7, and 5 participants for the control, HWL red, and HWL yellow groups did not answer any of the questions for the primary outcome; thus, data for 610 participants were available for the intention-to-treat analysis (Fig. 2).

Table 2 shows the characteristics of the study sample by intervention groups. The median age was 21, with more females in the study sample (65.4%). Over 60% of the participants pursued a bachelor’s degree, and around a quarter of the sample studied biological and health sciences. A total of 40% of the study sample reported consuming alcohol at least once a month. The study sample characteristics were generally balanced across experimental groups, except for sex and educational level. A higher percentage of females in the HWL yellow group was observed.

**Table 2:** Sociodemographic characteristics of the study sample^a^

Characteristics	Total *n* = 610	Control *n* = 193	HWL red *n* = 209	HWL yellow *n* = 208
Age, median (interquatile range)	21 (20–24)	22 (20–25)	21 (19–24)	21 (20–23)
Sex				
Female	65.4 (399)	59.1 (114)	65.1 (136)	71.6 (149)
Men	34.6 (211)	40.9 (79)	34.9 (73)	28.4 (59)
Educational level				
Pursuing a bachelor’s degree	67.8 (414)	62.1 (120)	68.4 (143)	72.6 (151)
Pursuing a postgraduate degree	30.7 (187)	36.3 (70)	30.6 (64)	25.5 (53)
Not responding	1.5 (9)	1.6 (3)	1.0 (2)	1.9 (4)
Field of specialization				
Physic mathematics and engineering	8.5 (52)	9.8 (19)	6.7 (14)	9.1 (19)
Biological and health sciences	23.3 (142)	26.9 (52)	22.6 (47)	20.7 (43)
Social sciences	12.0 (73)	13.0 (25)	11.5 (24)	11.5 (24)
Arts and humanities	3.9 (24)	3.1 (6)	5.3 (11)	3.4 (7)
Not applicable/ Not responding	52.3 (319)	47.2 (91)	53.9 (113)	55.3 (115)
Educational and employment status				
Studying	17.5 (107)	20.2 (39)	17.7 (37)	14.9 (31)
Studying and working	23.7 (144)	24.4 (47)	23.4 (49)	23.1 (48)
Studying and looking for a job	7.9 (48)	8.8 (17)	6.7 (14)	8.2 (17)
Not responding	50.9 (311)	46.6 (90)	52.2 (109)	53.8 (112)
Region				
North central	0.7 (4)	1.0 (2)	-	1.0 (2)
Northwest	9.0 (55)	7.2 (14)	9.6 (20)	10.1 (21)
Northeast	-	-	-	-
Western	10.5 (64)	10.4 (20)	8.1 (17)	13.0 (27)
Center	24.7 (151)	28.0 (54)	25.8 (54)	20.7 (43)
Mexico City	19.7 (120)	18.7 (36)	17.2 (36)	23.0 (48)
South central	15.9 (97)	17.6 (34)	16.3 (34)	13.9 (28)
South	18.5 (113)	16.1 (31)	21.1 (44)	18.3 (38)
Not responding	1.0 (6)	1.0 (2)	1.9 (4)	-
Socioeconomic status				
Low	35.1 (214)	36.8 (71)	31.2 (65)	37.5 (78)
Middle	31.6 (193)	31.6 (61)	34.4 (72)	28.4 (59)
High	33.3 (203)	31.6 (61)	34.4 (72)	34.1 (71)
Frequency of consumption of alcohol consumption				
At least once a week	20.4 (124)	17.6 (34)	19.6 (41)	23.6 (49)
At least once a month	40.3 (246)	42.5 (82)	40.2 (84)	38.4 (80)
At least once in the last 12 months	38.0 (232)	37.8 (73)	39.2 (82)	37.0 (77)
Not responding	1.3 (8)	2.1 (4)	1.0 (2)	1.0 (2)
Number of drinks drunk in only one occasion	4 (2–8)	4 (2–8)	4 (2–7)	4 (2–8)

*HWL red* Health warning label in red

*HWL yellow* Health warning label in yellow

^a^Values are %(n), unless otherwise indicated

Table 3 shows the distribution of responses to the main questions in the total sample and by study group. The highest prevalence of thinking somewhat the health risks of drinking the beer can was in the HWL red group, followed by the HWL yellow group. Also, the prevalence of thinking very much about the health risks of drinking the alcoholic beverage presented was higher in the intervention groups compared to the control group. The prevalence of participants that found the beer can attractive or very attractive was higher in the control versus the intervention groups. The highest prevalence of avoiding seeing the label was higher in the HWL red group, followed by the control group. More participants in the control group versus the intervention groups responded as likely or very likely they would buy the beer can. Unlike the previous result, more participants in the HWL red group responded as somewhat likely or likely they would consume the beer can, but more participants in the control group responded as very likely they would consume the alcoholic beverage presented.

**Table 3:** Distribution in responses on health warning labels according to intervention groups

Questions	Total	Control	HWL red	HWL yellow
	% (n)	% (n)	% (n)	% (n)
***Did you think about the health risks involved in drinking it?***				
Not at all	27.7 (169)	38.4 (74)	21.1(44)	24.5 (51)
A little	28.4 (173)	25.9 (50)	28.2 (58)	30.8 (64)
Somewhat	27.7 (169)	23.8 (46)	32.5 (68)	26.4 (55)
Very much	15.6 (95)	11.4 (22)	17.7 (37)	17.3 (36)
I do not wish to reply	0.6 (4)	0.5 (1)	0.5(1)	1.0 (2)
***How attractive do you find the product shown?***				
Not attractive at all	23.6 (144)	19.2 (37)	28.2 (59)	23.0 (48)
Little attractive	50.7 (309)	47.2 (91)	46.9 (98)	57.7 (120)
Attractive	22.4 (137)	26.9 (52)	23.0 (48)	17.8 (37)
Very attractive	3.1 (19)	6.7 (13)	1.9 (4)	1.0 (2)
I do not wish to reply	0.2 (1)	0.0 (0)	0.0 (0)	0.5 (1)
***Did you avoid seeing the label?***				
Yes	17.1 (104)	17.6 (34)	19.6 (41)	14.0 (29)
No	82.1 (501)	81.4 (157)	80.4 (168)	84.6 (176)
I do not wish to reply	0.8 (5)	1.0 (2)	0.0 (0)	1.4 (3)
***How likely are you to buy it?***				
Very unlikely	30.6 (187)	28.5 (55)	34.0 (71)	29.3 (61)
Unlikely	26.4 (161)	25.9 (50)	23.4 (49)	29.8 (62)
Somewhat likely	26.4 (161)	26.4 (51)	26.8 (56)	26.0 (54)
Likely	12.8 (78)	14.0 (27)	12.9 (27)	11.5 (24)
Very likely	3.8 (23)	5.2 (10)	2.9 (6)	3.4 (7)
I do not want to answer	-	-	-	-
***How likely are you to consume it?***				
Very unlikely	28.5 (174)	29.0 (56)	27.3 (57)	29.3 (61)
Unlikely	25.6 (156)	23.3 (45)	23.4 (49)	29.8 (62)
Somewhat likely	25.9 (158)	25.4 (49)	28.2 (59)	24.0 (50)
Likely	14.8 (90)	15.6 (30)	16.3 (34)	12.5 (26)
Very likely	5.0 (31)	6.7 (13)	4.8 (10)	3.9 (8)
I do not wish to reply	0.2 (1)	0.0 (0)	0.0 (0)	0.5 (1)

*HWL red* Health warning label in red

*HWL yellow* Health warning label in yellow

Table 4 shows the results of the models to assess the capacity of warning labels on thinking about health risks, product attractiveness, visual avoidance, and intention to change consumption. More participants in the intervention groups thought about the health risks associated with drinking the beer presented than in the control group [Prevalence ratio (PR) 1.43, 95% confidence interval (CI) 1.05,1.93 for the HWL red group; PR 1.25, 95%CI 0.91, 1.71 for the HWL yellow group]. We also observed that a lower percentage of young adults in the intervention groups versus the control group considered the product attractive, being statistically for the HWL yellow group (PR 0.74, 95%CI 0.51, 1.06 for the HWL red group; PR 0.56, 95%CI 0.38, 0.83 for the HWL yellow group). Although not statistically significant, a higher percentage of participants in the HWL red group avoided seeing the label, while a lower percentage in the HWL yellow group did the same compared to the control group. Also, a lower percentage of participants in the intervention groups (especially in the HWL yellow group) considered buying the product than the control group. The PR for buying the product was 0.93 (95%CI 0.70, 1.25) in the HWL red group and 0.90 (95%CI 0.67, 1.21) in the HWL yellow group. Neither was statistically significant the results about the likelihood of consuming the product; however, we also observed a lower prevalence of participants in the HWL yellow group versus in the control group that considered consuming the beer can [PR 0.85, (95%CI 0.63, 1.14)]. All results were similar when the models were adjusted for covariates.

**Table 4:** Estimated differences in the prevalence of responses to primary measures between study groups

Primary measures	Prevalence	Unadjusted model		Adjusted model ^a^	
		**PR**	**CI 95%**	**PR**	**CI 95%**
***Thought about the health risks involved in drinking it***		*n* = 606		*n* = 606	
Group I: Control	35.4	REF		REF	
Group II: Health warning label in red	50.7	1.43	(1.05, 1.93)	1.4	(1.03, 1.91)
Group III: Health warning label in yellow	44.2	1.25	(0.91, 1.71)	1.28	(0.93, 1.76)
***Attractive product***		*n* = 605		*n* = 605	
Group I: Control	33.7	REF		REF	
Group II: Health warning label in red	24.9	0.74	(0.51, 1.06)	0.76	(0.53, 1.1)
Group III: Health warning label in yellow	18.8	0.56	(0.38, 0.83)	0.58	(0.38, 0.86)
***Avoid seeing the label***		*n* = 605		*n* = 605	
Group I: Control	17.8	REF		REF	
Group II: Health warning label in red	19.6	1.10	(0.70, 1.74)	1.13	(0.71, 1.79)
Group III: Health warning label in yellow	14.1	0.79	(0.48, 1.3)	0.79	(0.48, 1.31)
***Likely to purchase***		*n* = 610		*n* = 610	
Group I: Control	45.6	REF		REF	
Group II: Health warning label in red	42.6	0.93	(0.70, 1.25)	0.94	(0.7, 1.26)
Group III: Health warning label in yellow	40.9	0.90	(0.67, 1.21)	0.88	(0.65, 1.19)
***Likely to consume***		*n* = 609		*n* = 609	
Group I: Control	47.7	REF		REF	
Group II: Health warning label in red	49.3	1.03	(0.78, 1.37)	1.04	(0.78, 1.38)
Group III: Health warning label in yellow	40.6	0.85	(0.63, 1.14)	0.85	(0.63, 1.15)

*PR* Prevalence ratio

*95%CI* 95% confidence interval

^a^Models adjusted for socioeconomic status, sex, age, education level, area of concentration of university studies, frequency of alcohol consumption and occupation

**Fig. 3:**
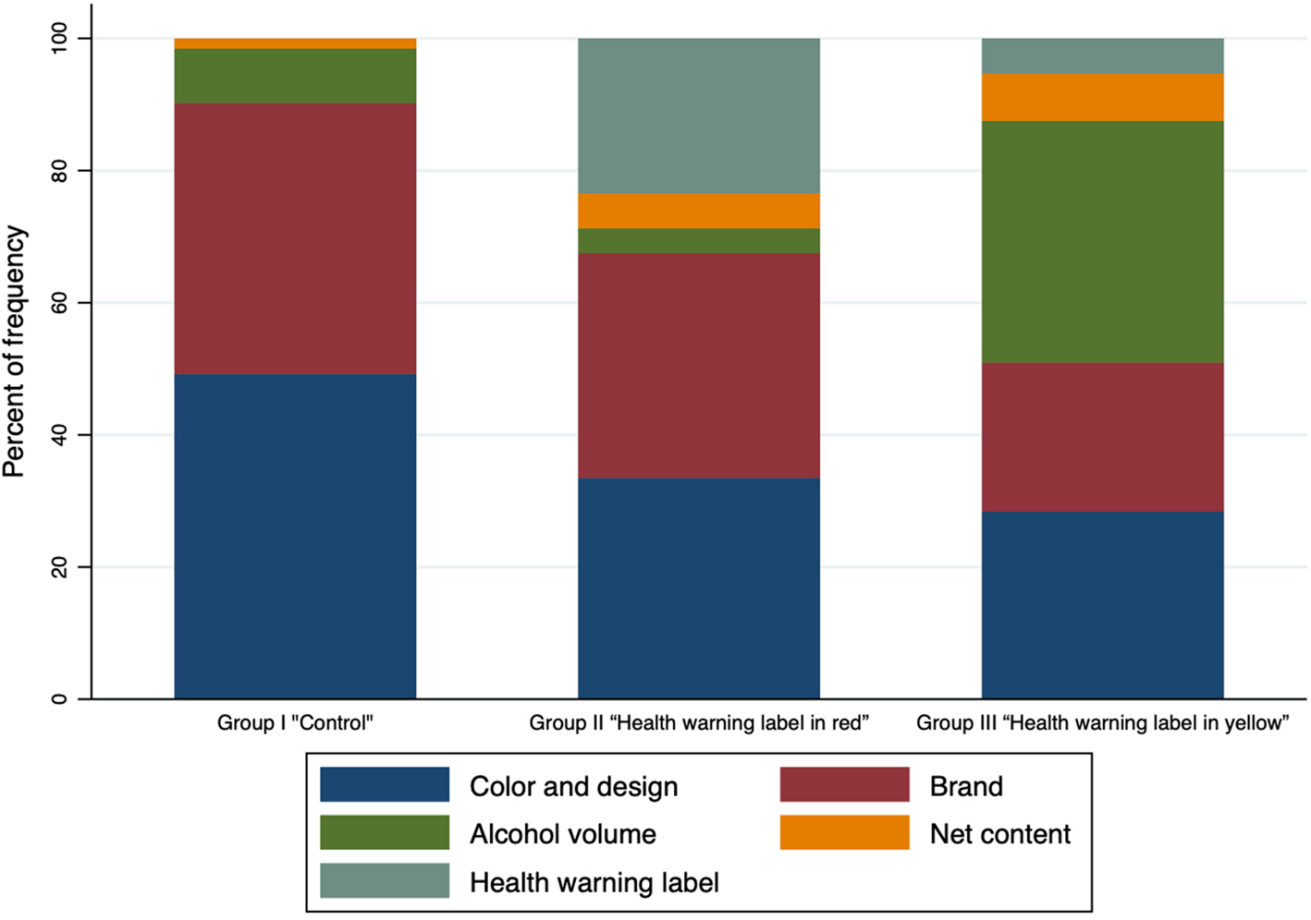
Characteristics of the beer can that attracted the most attention by experimental condition

Figure 3 shows the results of the beer can characteristics that attracted the most attention from participants. In the control group, the characteristics that most attracted attention were the color and design of the beer can (49.2%). In the HWL red group, 34.0% were more attracted by the color and design, and 23.4% by the warning label. In the HWL yellow group, 36.5% were more attracted by the volume of alcohol, and the warning label attracted only 5.3%.

When we analyzed the health warning messages grouped by type, we found that a higher percentage of participants who observed messages about mental health problems thought about the health risks involved in drinking the product compared to those who observed messages related to prohibitions on sale and consumption. Although not statistically significant, we also observed that a lower percentage of subjects who observed messages about health risks and mental health problems considered the product less attractive compared to those who observed messages related to prohibitions on sale and consumption. The prevalence of avoiding seeing the label was 82% higher in participants who observed messages related to mental health problems compared to those who observed messages related to prohibitions on sale and consumption (PR 1.82; 95%CI 1.00, 3.31) (Table 5).

**Table 5:** Estimated differences in the prevalence of responses to primary measures between study groups by type of health warning message

Primary measures	Unadjusted model		Adjusted model^a^	
	**PR**	**CI 95%**	**PR**	**CI 95%**
***Thought about the health risks involved in drinking it***	*n* = 414		*n* = 414	
Prohibitions on sale and consumption	REF		REF	
Health risks	1.02	(0.73,1.42)	1	(0.72,1.40)
Mental health problems	1.16	(0.81,1.66)	1.16	(0.80,1.68)
***Attractive product***	*n* = 416		*n* = 416	
Prohibitions on sale and consumption	REF		REF	
Health risks	0.75	(0.47,1.22)	0.73	(0.45,1.20)
Mental health problems	0.86	(0.51,1.45)	0.81	(0.47,1.40)
***Avoid seeing the label***	*n* = 414		*n* = 414	
Prohibitions on sale and consumption	REF		REF	
Health risks	1.04	(0.58,1.87)	1.15	(0.73,1.84)
Mental health problems	*1.73*	*(0.97,3.08)*	1.82	(1.00,3.31)
***Likely to purchase***	*n* = 417		*n* = 417	
Prohibitions on sale and consumption	REF		REF	
Health risks	0.76	(0.53,1.08)	0.75	(0.52,1.09)
Mental health problems	1.02	(0.70,1.47)	0.97	(0.66,1.42)
***Likely to consume***	*n* = 416		*n* = 416	
Prohibitions on sale and consumption	REF		REF	
Health risks	0.85	(0.61,1.20)	0.83	(0.58,1.17)
Mental health problems	1.02	(0.76.1. 55)	0.99	(0.68,1.44)

*PR* Incidence rate-ratio

*95%CI* 95% confidence interval

^a^Models adjusted for socioeconomic status, sex, age, education level, area of concentration of university studies, frequency of alcohol consumption and occupation

## Discussion

We evaluated the potential impact of health warning labels on thinking about health risks, attitudes, and intention to change alcohol consumption among young adults in Mexico. We found that participants exposed to a can of beer with a health warning label were more thoughtful about the health risks of drinking beer than the control group. Also, a lower percentage of young adults in intervened vs control group considered the product attractive, especially among those observing the health warning label in yellow. Although not statistically significant, more participants in the health warning label in yellow mentioned they avoided seeing the label. Neither was statistically significant, but a lower percentage of participants in the intervention groups, especially those who observed a yellow health warning label, considered buying or consuming the product than the control group. Our post-hoc analyses showed that participants assigned to health risks and mental health messages thought more frequently about the health risks involved in drinking than those who observed messages about prohibitions on sale and consumption.

Our findings related to thinking about health risks are consistent with prior studies that found higher frequency of thinking about alcohol health risks alcohol in the groups exposed to health warning labels compared to controls [18, 19, 28, 29]. Increasing the thoughts of health risks from consuming alcoholic beverages is relevant. First of all, consumers have the right to know the potential harms related to alcohol consumption. Also, even though information-based interventions might not be sufficient to change population-level behaviors on their own or for implementation issues, they are necessary to educate the public about why changing the behavior is important and encourage support to implement other strategies affecting behaviors, such as taxation and advertising restrictions [12, 30].

Fewer participants in the intervention groups found the can of beer attractive, suggesting that exposure to warning labels can lead to developing negative attitudes toward the product [14]. Similar results have been observed for sugar-sweetened beverages and tobacco [14, 21]. Whether the negative attitudes translate into on behaviors is beyond the scope of our study, and will need to be further explored. The latter is especially relevant considering that fewer participants in the yellow health warning than in the control group considered the product attractive, but more participants in this group avoided seeing the label. Based on previous studies on sugar-sweetened beverages and tobacco, this double negative attitude may reflect more a defensive avoidance with a potential negative impact on behaviors [16]. On the other hand, a lower number of participants in the red health warning label group than in the control group also found the product attractive, but the proportion that avoided seeing the label was similar to that observed in the control group. This combination of reactions might eventually conduct in less preference for the product [14]. These hypotheses will be needed to test in future studies.

Although we did not find statistically significant differences in the intention to consume or purchase across experimental groups, we did observe a lower percentage of participants in the intervention groups with the intention to consume or purchase the beer can presented. The results of the potential effect of warning labels on intent to purchase and consume alcoholic beverages have been inconsistent across studies. Clarke, et al., experimented in a naturalistic shopping lab (a space with similar characteristics to a supermarket) and found that the selection of alcoholic beverages was not different between study groups [19]. Krischler & Glock, in a laboratory study, found no difference between intervention groups in terms of intention to consume alcoholic beverages [31]. In their virtual experimental studies, Ma and Pettigrew, et al., also failed to observe differences in intention to consume alcohol between study groups [32]. On the contrary, Jongenelis et al. and Wigg & Stafford found greater intent to reduce alcohol consumption in groups of participants who observed some form of health warning labeling compared to the control group [29, 33].

That studies find no difference or differences of great magnitude in the intention to change in purchases and consumption of alcohol may be an expected result in studies where participants are exposed only once to warning messages. The study by Zhao et al., the largest real-world experiment conducted until now, found a reduction in per capita alcohol sales after 14-month exposure to health warning labels with rotating health and safety messages as compared to control sites [34]. This finding highlights the importance of exposing populations to warning labels over time, to introduce them to a process of contemplation and subsequent elaboration of thoughts about the consequences of continuing with a specific behavior [35].

As an exploratory analysis, we also evaluated the potential effect of groups of messages on participant's responses. The results showed that health warning messages associated with mental health problems seemed to have a greater impact on thinking about health risks and avoiding seeing the label than those related to banning the sale or consumption of alcohol. Currently in Mexico, only the pictogram related to prohibiting the sale of alcohol to minors is mandatory in beer cans [9]. However, it is likely that people who consume beer also consume other alcoholic beverages (alone or in combination), where pictograms related to banning alcohol consumption while driving or during pregnancy are mandatory. Therefore, it might be expected that health warning messages related to banning alcohol consumption, especially about prohibiting the sale to minors, have a low impact, not only because the participants might have seen them before in alcohol containers but also in retail stores [36]. On the other hand, health warning messages associated with mental health problems might have a higher impact on young adults because this was the first time they observed specific health risk messages in an alcohol container. Moreover, information about mental health risks might be more meaningful for young adults [37]. Future studies will be needed to properly evaluate each type of warning message’s impact.

Our study has limitations including not reaching the expected sample size, which could explain why we did not detect some differences in outcomes between study groups. Although the study sample was balanced for most of the variables collected, random differences might remain given that we used simple randomization and the sample size was insufficiently large. Also, the representativeness is limited to students with upper-middle and upper-level education and access to internet. We could also assume that the study sample better represents students of upper-middle education in whom a field of specialization is not expected and in whom the educational and employment status was not asked by an error in the online questionnaire. Further studies are required to determine the consistency of findings in populations with more diverse characteristics (e.g., in populations with lower educational levels) and in environments closer to reality (e.g., purchasing laboratories). Likewise, it will be necessary to replicate the study in a larger study sample to evaluate: 1) which type of labeling (red or yellow) is more suitable for the population in Mexico, 2) the use of symbols versus authentic images (such as those presented on cigarette packs), 3) the understanding of warning messages and 4) the deterrence capacity of different warning messages. Finally, we cannot rule out the possibility that the impact of health warning labels is different in other alcoholic beverages. We focused on studying the potential impact of health warning labels on beer cans among consumers of this type of beverage not only because it is the alcoholic beverage most consumed in Mexico but also to avoid that preferences could influence the estimations.

## Conclusions

Our findings, in line with prior research, suggest that health warning labels designed to be visible can lead to individuals thinking about the health risks of alcohol and reducing the attractiveness of the product. The results also highlight the potential of health warning labels to reduce the intention to purchase and consume alcohol. Given the nature of this one-time online experiment, the findings about thinking about the health risks of consuming alcoholic beverages are particularly relevant since this is a needed process for future behavior changes. Further studies are still needed to determine which pictograms and legends are most suitable in Mexico and other countries, considering the diversity in sociodemographic and cultural contexts.

## Supplementary Information

**Additional file 1: Supplementary Figure 1. **Design of beer cans used according to the intervention group. **Supplementary Table 1. **Selection of states by geographic region.

## Data Availability

Data at the participant level used during the current study are not publicly available due to a lack of ethical approval for the data-sharing policy. Any other aggregated data not presented in this study are available from the corresponding author upon reasonable request.

## Abbreviations

95%CI: 95% Confidence interval
CISIDAT: Consorcio de Investigación en Salud
HWL red: Health warning label in red
HWL yellow: Health warning label in yellow
PR: Prevalence ratio
WHO: World Health Organization
