# Supplementary material for: Warning labels on alcoholic beverage containers: a pilot randomized experiment among young adults in Mexico
Source: BMC Public Health. 2023 Jun 15;23:1156. doi: 10.1186/s12889-023-16069-w (PMC10268389; doi:10.1186/s12889-023-16069-w)
Supplement: Supplementary file 1 — Additional file 1: Supplementary Figure 1. Design of beer cans used according to the intervention group. Supplementary Table 1. Selection of states by geographic region. [file 12889_2023_16069_MOESM1_ESM.docx]

**Supplementary material**

**Supplementary Figure 1. Design of beer cans used according to the intervention group.**

| **Group II. Health warning labels in red.** | **Group III. Health warning labels in yellow.** |
| --- | --- |
| 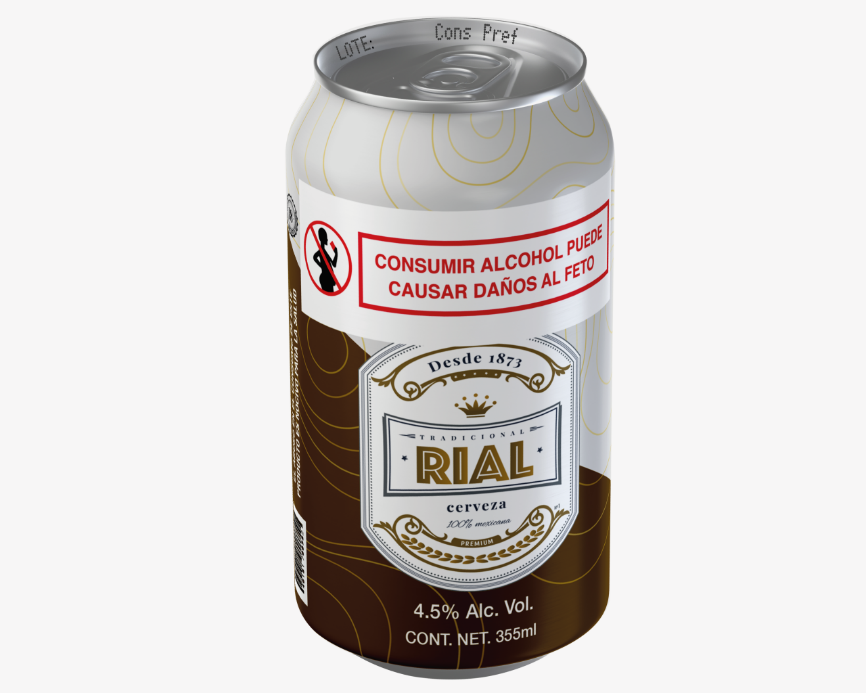 | 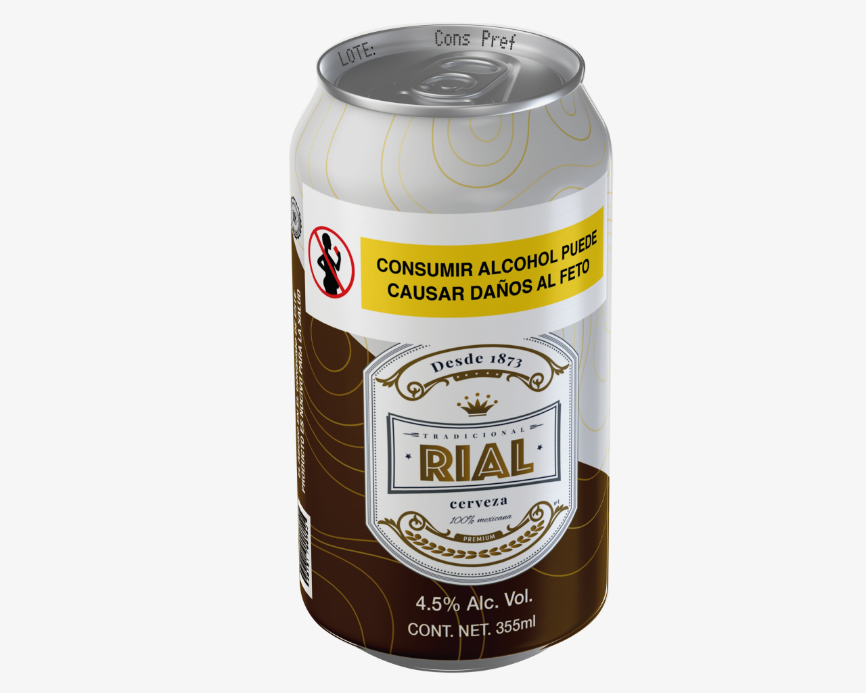 |
| 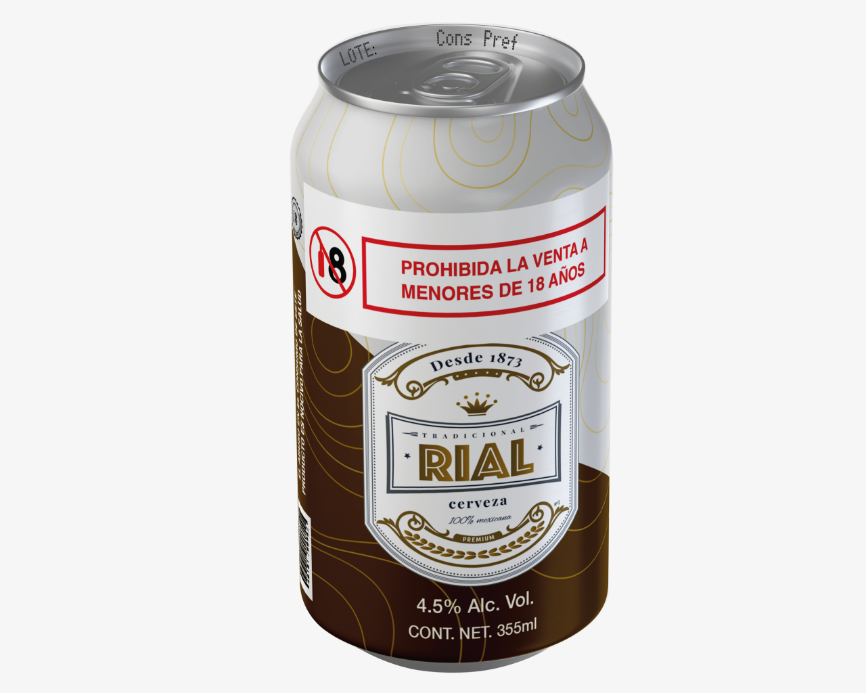 | 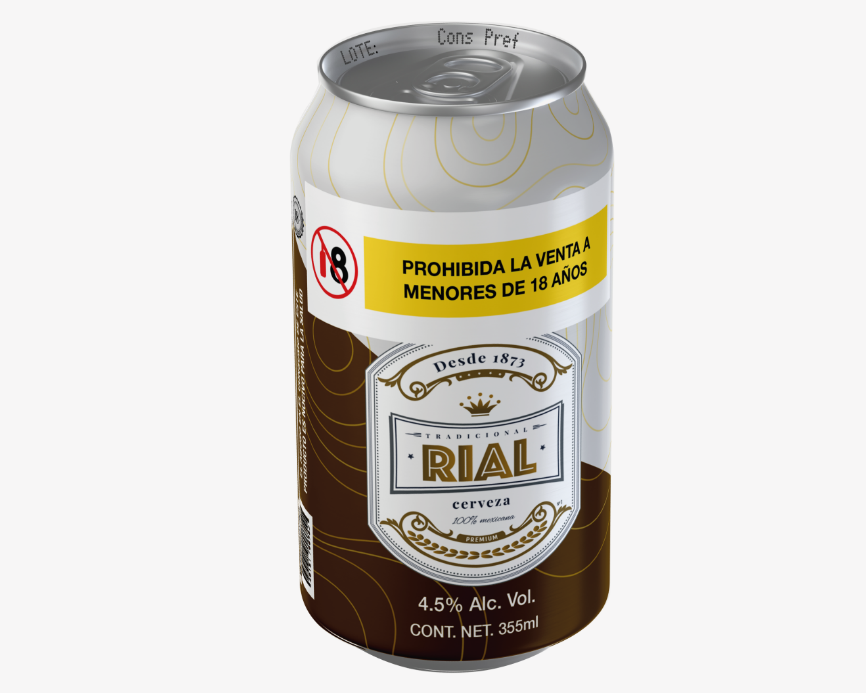 |
| 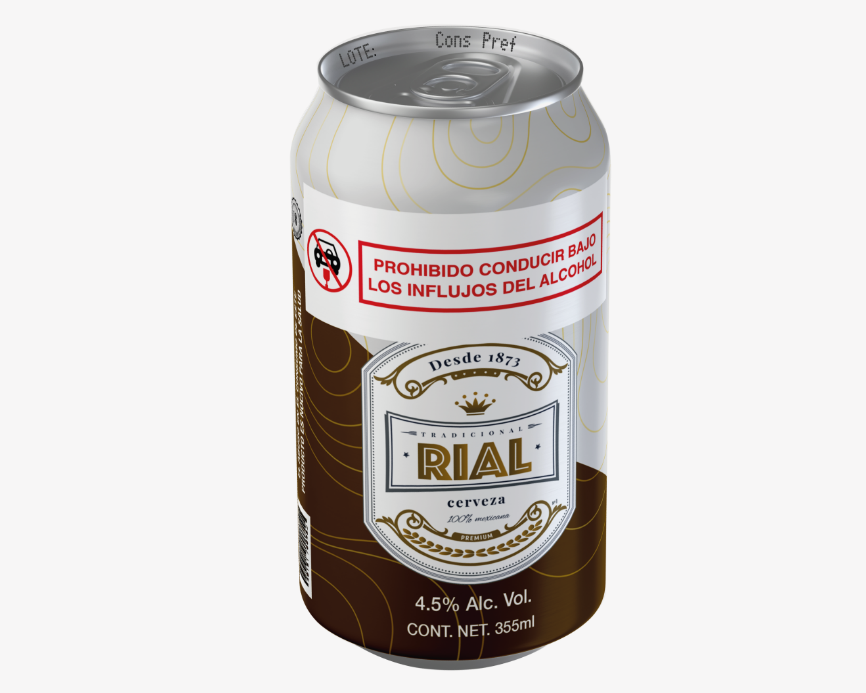 | 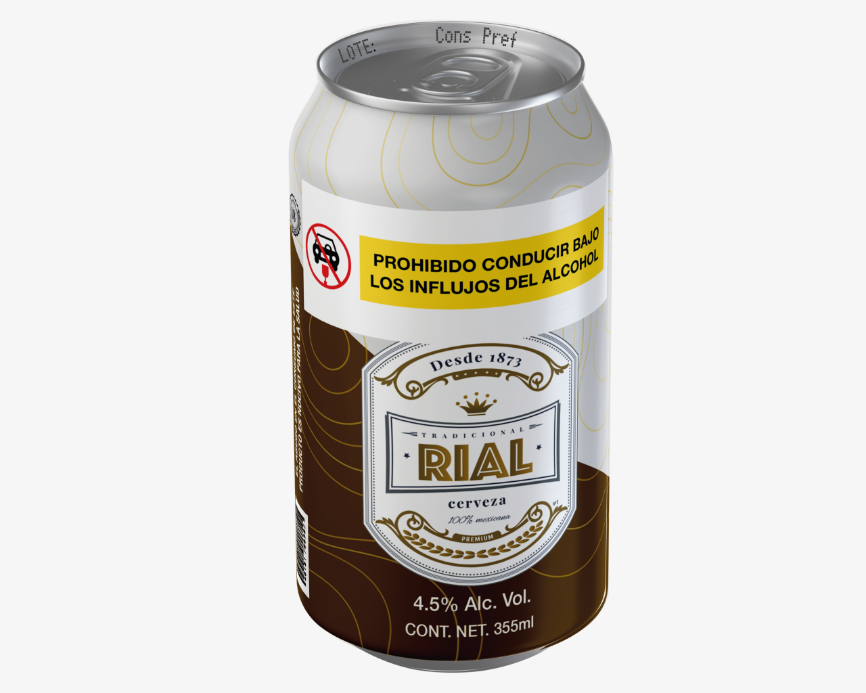 |
| 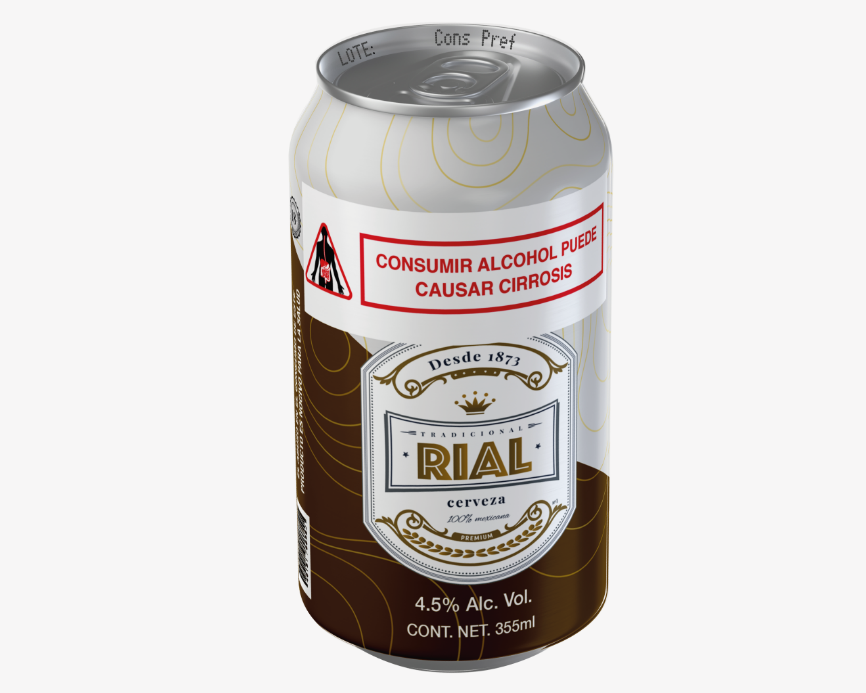 | 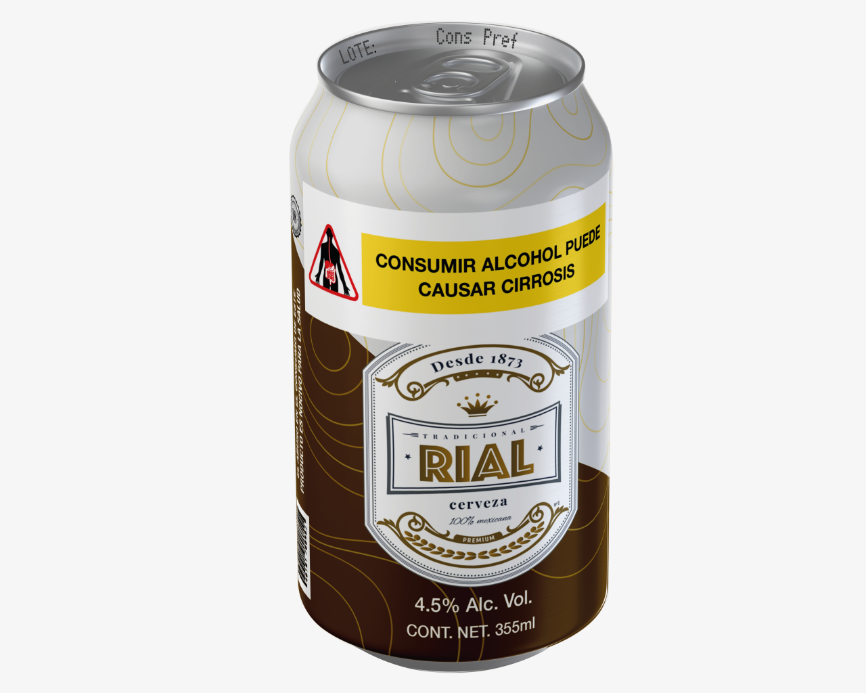 |
| 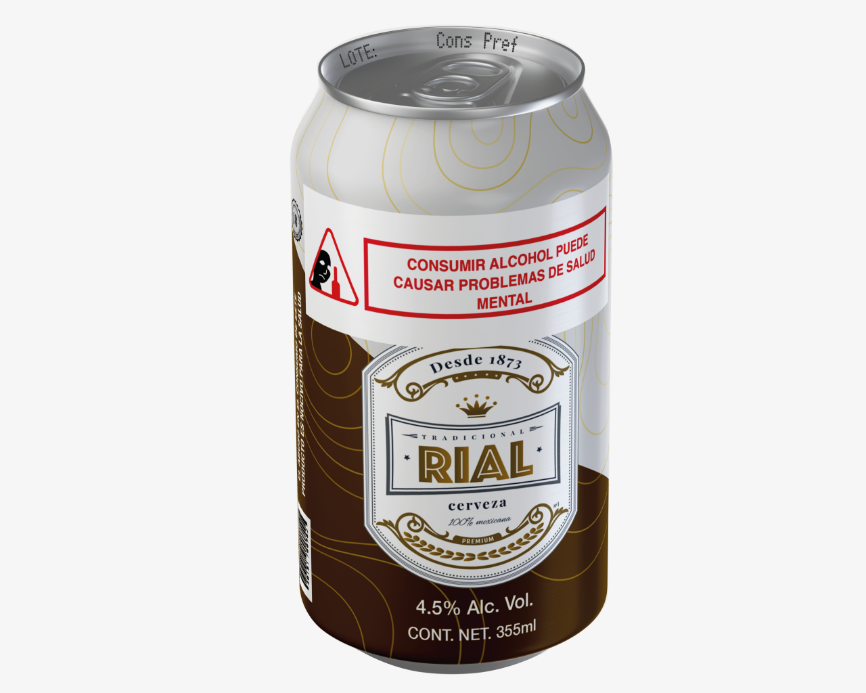 | 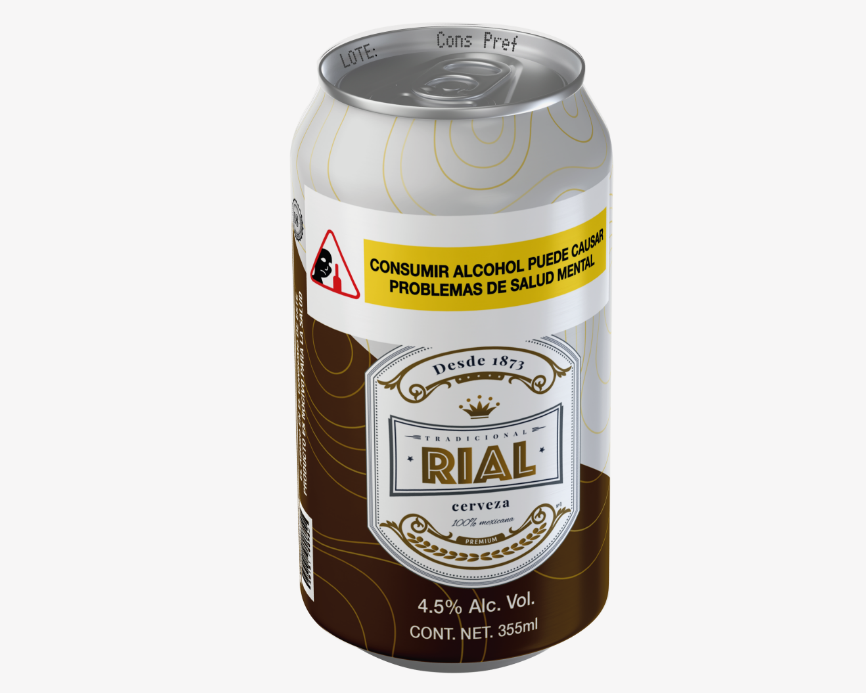 |
| 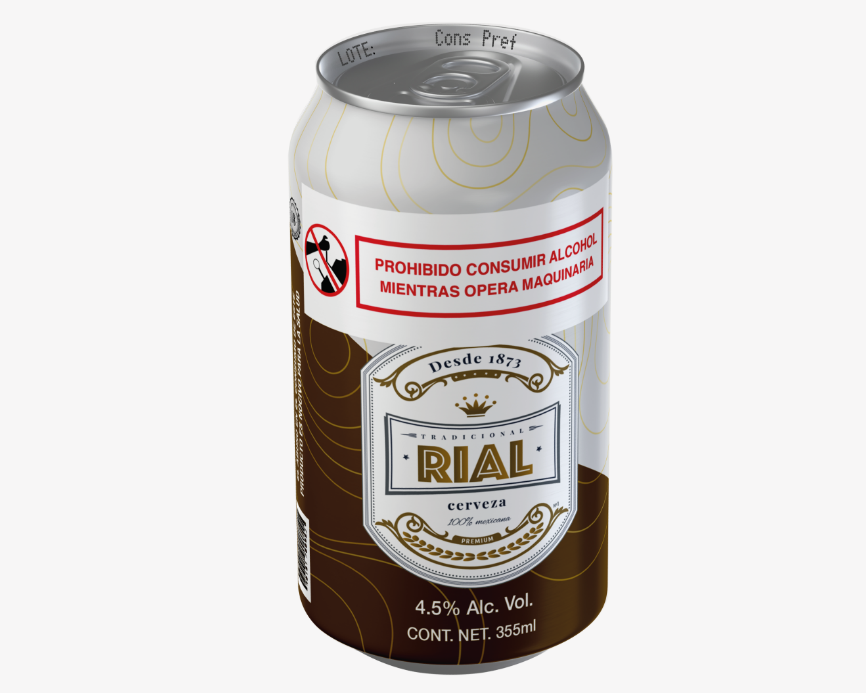 | 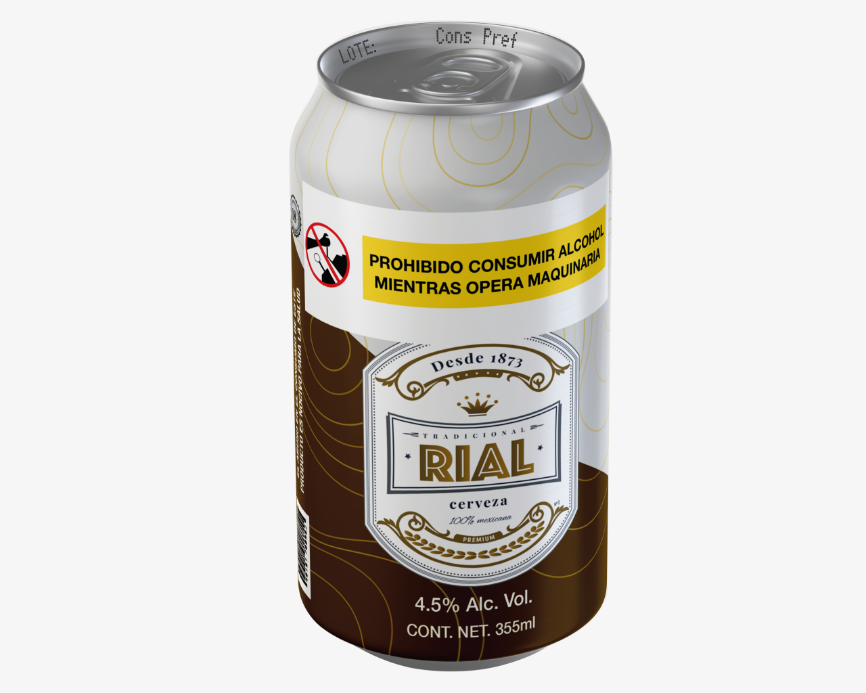 |
| 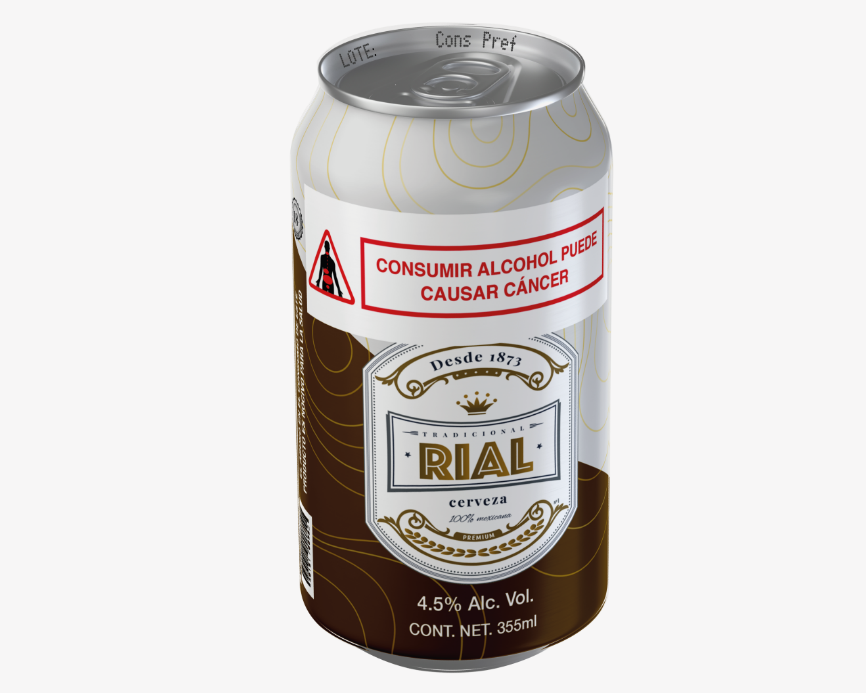 | 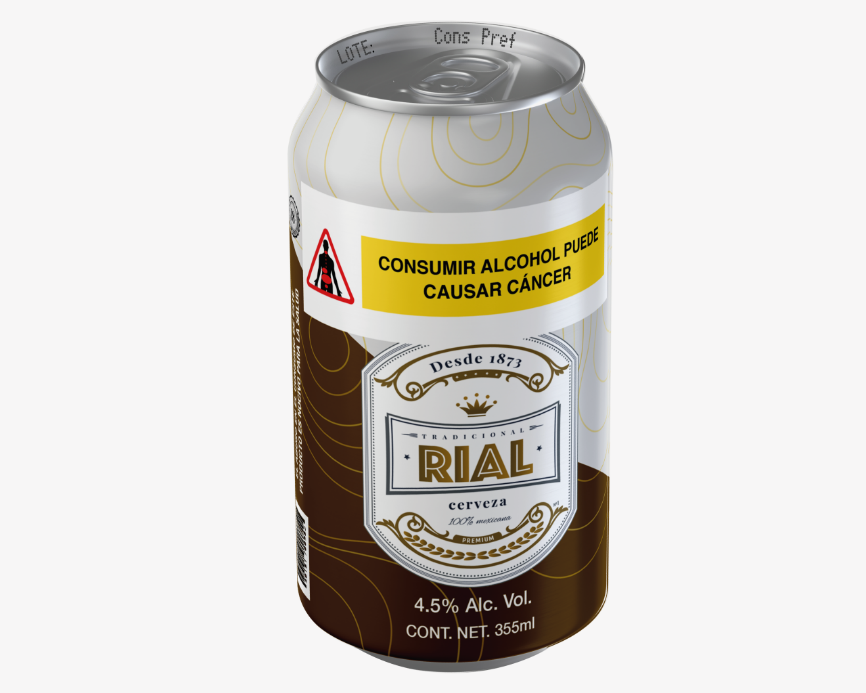 |
| 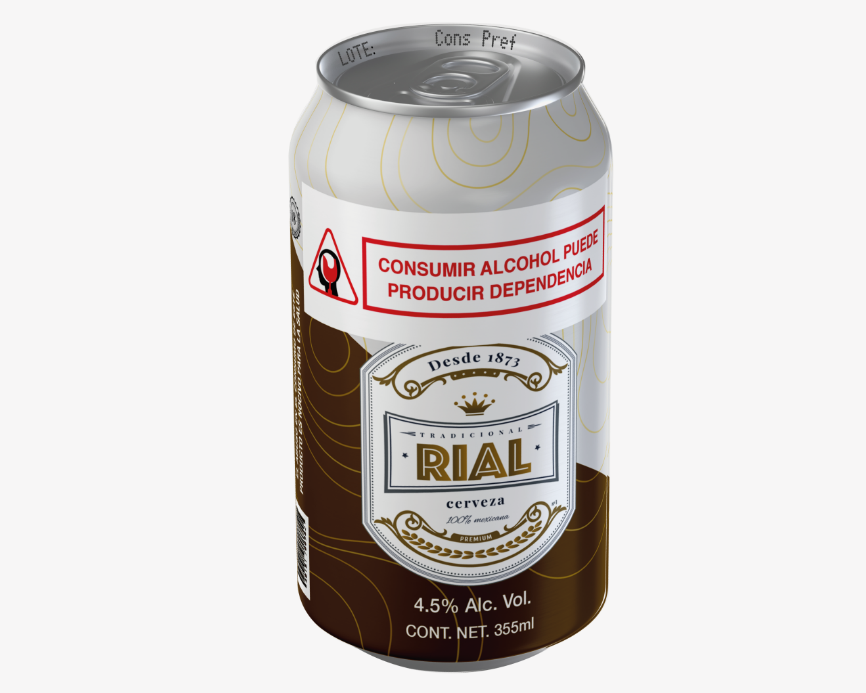 | 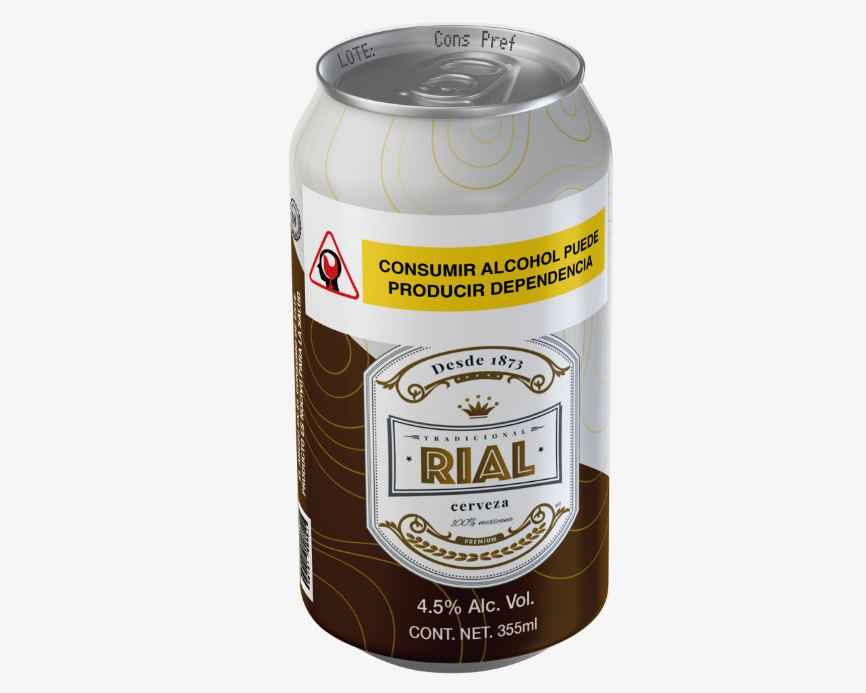 |

**Supplementary Table 1. Selection of states by geographic region.**

| **Región** | **State** |
| --- | --- |
| North-central | Durango |
| Northwestern | Baja California Sur |
| Northeastern | Tamaulipas, |
| West | Zacatecas |
| Central | Morelos, Estados de México,  Guanajuato |
| Mexico City | Mexico City |
| Central-South | Veracruz, Guerrero |
| South | Yucatán |
